# Supplementary material for: Toward sub-second solution exchange dynamics in flow reactors for liquid-phase transmission electron microscopy
Source: Nat Commun. 2024 Mar 21;15:2522. doi: 10.1038/s41467-024-46842-3 (PMC10957994; doi:10.1038/s41467-024-46842-3)
Supplement: Supplementary file 1 — Supplementary Information [file 41467_2024_46842_MOESM1_ESM.pdf]

– Supplementary Information –

Toward sub-second solution exchange dynamics in flow reactors for liquid-phase transmission electron microscopy

Author list

Stefan Merkens,[1,2] Christopher Tollan,[1] Giuseppe De Salvo,[1,2] Katarzyna Bejtka,[3,4] Marco Fontana,[3,4] Angelica Chiodoni,[3] Joscha Kruse,[1,5] Maiara Aime Iriarte-Alonso,[1,6] Marek Grzelczak,[5,7] Andreas Seifert,[1,8] Andrey Chuvilin[1,8]

Affiliations

1. CIC nanoGUNE BRTA, Tolosa Hiribidea 76, 20018 Donostia – San Sebastián, Spain
2. Department of Physics, Euskal Herriko Unibertsitatea (UPV/EHU), 20018 Donostia – San Sebastián, Spain
3. Center for Sustainable Future Technologies@Polito, Istituto Italiano di Tecnologia (IIT), Via Livorno, 60, 10144 Torino TO, Italy
4. Department of Applied Science and Technology (DISAT), Politecnico di Torino, corso Duca degli Abruzzi 24, 10129 Torino, Italy
5. Donostia International Physics Center (DIPC), Paseo Manuel de Lardizabal 4, 20018 Donostia – San Sebastián, Spain
6. TECNIPESA IDENTIFICACION SL, Parque Empresarial Zuatzu, Edificio Donosti 1-3, 20018 Donostia-San Sebastián, Spain
7. Centro de Física de Materiales CSIC-UPV/EHU, Paseo Manuel de Lardizabal 5, 20018 Donostia – San Sebastián, Spain
8. IKERBASQUE, Basque Foundation for Science, Plaza Euskadi 5, 48009 Bilbao, Spain

Corresponding author

Stefan Merkens (s.merkens@nanogune.eu)

Contents

Supplementary Note 1. Theoretic background 2

Supplementary Note 2. Prototyping of diffusion cells 6

Supplementary Note 3. Mass transport mechanisms in diffusion cell setups 8

Supplementary Note 4. Physical prototyping of ex-situ sample holder 18

Supplementary Note 5. Further scenarios and improved experimental methods 21

Supplementary Note 6. Application experiment 23

Supplementary Note 1. Theoretic background

Fundamental equations for single flow channel

The mean travel time, t, for convective transport follows from Eq. 1, where s is the travelled distance and $\bar{v}$ is the mean flow velocity:

$$t = s/\bar{v} \tag{1}$$

The volumetric flow rate, Q_{total}, is related to $\bar{v}$ through Eq. 2, where A is the channel cross-section:

$$Q_{\mathrm{total}} = \bar{v}A \tag{2}$$

The general definition of flow channel resistance, R, is provided in Eq. 3, where Δp denotes the pressure gradient along the flow channel:

$$R = \Delta p / Q_{\mathrm{total}} \tag{3}$$

Fundamental equations for multiple parallel flow channels

When multiple flow channel compartments are arranged in parallel, the total volumetric flow rate, Q_{total}, follows as the sum of the fractions Q_{i} passing through each segment (Eq. 4).

$$Q_{\mathrm{total}} = \sum_{\mathrm{i}} Q_{\mathrm{i}} \tag{4}$$

Inserting Eq. 4 in Eq. 3 results in an expression for the resistance of each channel compartment (Eq. 5):

$$R_{\mathrm{total}} = \frac{\Delta p}{Q_{\mathrm{total}}} = \frac{\Delta p}{\sum_{\mathrm{i}} Q_{\mathrm{i}}} \tag{5}$$

$$\frac{1}{R_{\mathrm{total}}} = \sum_{\mathrm{i}} \frac{1}{R_{\mathrm{i}}} \tag{6}$$

$$R_{\mathrm{i}} = \frac{\Delta p}{Q_{\mathrm{i}}} \tag{7}$$

Poiseuille plane flow

Poiseuille plane flow describes flow through channels with rectangular cross-section of high aspect ratio (width >> height). The parabolic velocity profile along the short channel dimension (y) through a Poiseuille plane is described by Eq. 10.

$$v(y) = -\frac{1}{2\mu}\left(\frac{\mathrm{d}p}{\mathrm{d}x}\right)(h^2 - y^2) \tag{8}$$

The mean and the maximum velocity in Poiseuille planes are related (Eq. 9.1). Following Eq. 8, $\bar{v}$ is defined by Eq. 9.2.

$$\bar{v} = \frac{2}{3} v_{\mathrm{max}} \tag{9.1}$$

$$= v(y = 0) = \frac{\mathrm{d}p}{\mathrm{d}x}\left(\frac{h^2}{3\mu}\right) \tag{9.2}$$

Convective transport in NC

The fundamentals of Eq. 1 – 9.2 can be applied to derive a dependence of the time constant, t_C, for convective transport in the nanochannel of diffusion cell configurations on accessible experimental parameters. By applying Eq. 1 and 2, assuming $R_{\mathrm{BP}} \ll R_{\mathrm{NC}}$, where R_{BP} combines both on- and off-chip bypass compartments (compare Supplementary Note 2), for t_C follows:

$$t_{\mathrm{C}} = \frac{\left(\frac{w_{\mathrm{NC}}}{2}\right)}{\left(\frac{Q_{\mathrm{NC}}}{A_{\mathrm{NC}}}\right)} \tag{10.1}$$

$$= \frac{w_{\mathrm{NC}}^2}{2Q_{\mathrm{total}}}\left(\frac{R_{\mathrm{NC}}+R_{\mathrm{BP}}}{R_{\mathrm{BP}}}\right) \tag{10.2}$$

$$\approx \frac{w_{\mathrm{NC}}^2}{2Q_{\mathrm{total}}}\left(\frac{R_{\mathrm{NC}}}{R_{\mathrm{BP}}}\right) \tag{10.3}$$

$$= \frac{w_{\mathrm{NC}}^2}{2Q_{\mathrm{total}}}\left(\frac{Q_{\mathrm{total}}-Q_{\mathrm{NC}}}{Q_{\mathrm{NC}}}\right) \tag{10.4}$$

$$\approx \frac{w_{\mathrm{NC}}^2}{2Q_{\mathrm{NC}}} \tag{10.5}$$

With Eq. 2 and Eq. 9.2, one obtains:

$$= \frac{w_{\mathrm{NC}}^2}{2}\frac{(3\mu)}{\left(\frac{\mathrm{d}p}{\mathrm{d}x}\right)h_{\mathrm{NC}}^3 w_{\mathrm{NC}}} \tag{10.6}$$

$$= \frac{w_{\mathrm{NC}}}{2}\frac{3\mu}{\left(\frac{\mathrm{d}p}{\mathrm{d}x}\right)h_{\mathrm{NC}}^3} \tag{10.7}$$

With $\left(\frac{\mathrm{d}p}{\mathrm{d}x}\right) = \frac{\Delta p}{L} = \frac{\Delta p}{w_{\mathrm{NC}}}$, one obtains:

$$= \frac{3\mu}{2}\frac{w_{\mathrm{NC}}}{\left(\frac{\Delta p}{w_{\mathrm{NC}}}\right)h_{\mathrm{NC}}^3} \tag{10.8}$$

$$= \frac{3\mu}{2}\frac{w_{\mathrm{NC}}^2}{\Delta p\, h_{\mathrm{NC}}^3} \tag{10.9}$$

With Eq. 5 and $R_{\mathrm{BP}} \approx L = w_{\mathrm{NC}}$ and $Q_{\mathrm{BP}} \approx Q_{\mathrm{total}}$, it follows:

$$= \frac{3\mu}{2h_{\mathrm{NC}}^3} \frac{w_{\mathrm{NC}}^2}{R_{\mathrm{B}} Q_{\mathrm{B}}} \tag{10.10}$$

$$\frac{3\mu}{2h_{\mathrm{NC}}^3} \frac{w_{\mathrm{NC}}^2}{R_{\mathrm{BP}} Q_{\mathrm{BP}}} \tag{10.11}$$

$$\approx \frac{w_{\mathrm{NC}}^2}{w_{\mathrm{NC}} Q_{\mathrm{total}}} \tag{10.12}$$

Thus, t_{C} can finally be expressed as

$$t_{\mathrm{C}} \approx \frac{w_{\mathrm{NC}}}{Q_{\mathrm{total}}} \tag{10.13}$$

Flow channel resistance in LP-TEM flow systems with diffusion cells

When additional on-chip bypass compartments are included, the total flow resistance is affected. The total resistance, R_{PR}, in the direct flow with premixing setups with diffusion cells can be expressed by Eq. 11, following from symmetry considerations similar to those presented in Supporting Information of a previous manuscript:[1]

$$\frac{1}{R_{\mathrm{PR}}} \approx \left(\frac{2}{2R_{\mathrm{G}}+2R_{\mathrm{off}}} + \frac{1}{R_{\mathrm{NC}'}} + \frac{1}{2R_{\mathrm{on}}} + \frac{1}{2R_{\mathrm{on}}}\right) \tag{11.1}$$

$$\approx \left(\frac{2}{2R_{G}+2R_{\mathrm{off}}} + \frac{1}{R_{\mathrm{NC}'}} + \frac{2}{2R_{\mathrm{on}}}\right) \tag{11.2}$$

In Eq. 11, R_{G} and $R_{\mathrm{NC'}}$ correspond to the resistance of a leak channel at each individual gasket, the resistance of the central nanochannel with reduced expansion in horizontal plane. R_{off} and R_{on} denote the resistance that corresponds to one quarter of the entire off-chip and on-chip bypass channel, respectively. Note that R_{off} is equivalent to R_{BP} in Ref. [1]. $R_{\mathrm{BP,PR}}$ can thus be reassigned to further simplify Eq. 11.2:

$$\frac{1}{R_{\mathrm{PR}}} \approx \left(\frac{1}{R_{\mathrm{BP,PR}}} + \frac{1}{R_{\mathrm{NC}'}}\right), \tag{11.3}$$

where

$$\frac{1}{R_{\mathrm{BP,PR}}} = \frac{2(2R_{\mathrm{G}}+2R_{\mathrm{off}})+2(2R_{\mathrm{on}})}{(2R_{\mathrm{G}}+2R_{\mathrm{off}})2R_{\mathrm{on}}}. \tag{12}$$

The total resistance, R_{OS}, in the bathtub with on-site mixing configuration with diffusion cells can be approximated as follow: [1]

$$\frac{1}{R_{\mathrm{OS}}} \approx \left(\frac{4}{3R_{\mathrm{off}}} + \frac{1}{R_{\mathrm{NC'}}} + \frac{4}{3R_{\mathrm{on}}}\right). \tag{13.1}$$

Note that also in Eq. 13.1, R_{off} is equivalent to R_{BP} in Ref. [1]. $R_{\mathrm{BP,OS}}$ can thus be reassigned to further simplify Eq. 13.1:

$$\frac{1}{R_{\mathrm{OS}}} \approx \left(\frac{1}{R_{\mathrm{BP,OS}}} + \frac{1}{\mathrm{R_{NC'}}}\right), \tag{13.2}$$

where

$$\frac{1}{R_{\mathrm{BP,OS}}} = \frac{12R_{\mathrm{off}}+12R_{\mathrm{on}}}{144R_{\mathrm{on}}R_{\mathrm{off}}} = \frac{R_{\mathrm{off}}+R_{\mathrm{on}}}{12R_{\mathrm{on}}R_{\mathrm{off}}}. \tag{14}$$

In context of the virtual prototyping reported in Fig. 3, R_{G} and R_{BP} remain unchanged whereas R_{on} and $R_{\mathrm{NC'}}$ are varied. R_{on} scales with w_{NC} and inversely with the height of the on-chip bypass channel. $R_{\mathrm{NC'}}$ scales strongly with w_{NC} and is significantly reduced with respect to R_{NC} describing the default nanochannel with nominal extension of 2 × 2 mm^2. Note that $R_{\mathrm{NC'}}$ and R_{on} are related through w_{NC} – widening one compartment, shrinks the other. Refer to previous literature for the dependence of flow resistance on the channel geometry.[1]

Supplementary Note 2. Prototyping of diffusion cells

In-depth discussion of virtual prototyping (Fig. 3)

Find below an in-depth discussion of the effect of geometrical (w_{NC} and h_{BP}) and experimental (Q_{total}) parameters on the monitored hydrodynamic parameters ($\Delta p, v_c, \Delta t$ and τ) for direct flow with premixing and bathtub with on-site mixing configuration with diffusion cells depicted in Fig. 3. For theoretic background refer to Supplementary Note 1 and 2 and previous literature.[1]

a-c) The overall pressure gradient Δp decreases when additional on-chip BPs are inserted (**a** and **b**). This reflects the decrease of the overall flow resistance R_{total} with increasing w_{NC} and h_{BP} in both direct flow with premixing and bathtub with on-site mixing configuration with diffusion cells, respectively (compare Eq. 1). For identic on-chip BP dimensions, the Δp-values are larger for the premixing than for the on-site mixing configuration, due to the substantially blocked off-chip BP (gasket technology[1,2]) resulting in larger R_{total}. Therefore, the biggest difference is observed in the setups with default flow cell (red cross and blue circle). However, the difference between both configurations decreases with increasing size of the on-chip BP (refer to **b**). This mainly is because the flow resistance, R_{on}, of the on-chip BP increasingly dominates R_{total} as $1/R_{on}$ is growing with w_{NC}, and more significantly with h_{BP} (compare Eq. 11.1 and 13.1). Ultimately, limiting values of Δp are obtained with increasing w_{NC} (compare **a**) and eventually h_{BP}. The achievable minima are at larger Δp in **a** than in **b** due to stronger geometric limitations (width of the on-chip BP: $w_{on} = 2$ mm – w_{NC} with $0 < w_{NC} < 2$). The cross-section, A, of the on-chip BP in **a** ($A = w_{on} \cdot h_{BP} = 1.95\ \text{mm} \cdot 10\ \mu\text{m} = 0.0195\ \text{mm}^2$) is restricted to significantly smaller values compared to those achievable by increasing h_{BP} ($A = w_{on} \cdot h_{BP} = 1.95\ \text{mm} \cdot 50\ \mu\text{m} = 0.09\ \text{mm}^2$ for the last point in **b**).

Δp further increases with the applied overall flow rate, Q_{total} (**c**). This reflects the direct proportionality between both quantities as follows from Eq. *2* and 3.

d-f) The dependence of v_c on w_{NC} and h_{BP} largely resembles that of Δp (**a** and **b**). The majority of the flow is guided through the available bypass channels and around the central nanochannel due to its relatively large resistance. In parallel assemblies, fractions of Q_{total} are redistributed in the different channel compartments based on their relative flow resistance, R_i (compare Eq. 7). In fact, the similarities between v_c and Δp are more pronounced for the premixing configuration, reflecting its direct flow characteristics with flow symmetrically passing the central nanochannel on both sides (compare Fig. 1). In contrast, v_c in on-site mixing configuration with diffusion cell displays slight increase with decreasing $w_{NC} < 1.8$ mm and at intermediate h_{BP} of 5 to 10 µm. The increase with decreasing w_{NC} (**d**) and at intermediate h_{BP} is due to decreasing flow resistance of the central nanochannel and flow being redirected closer to the NC of lower resistance (*vs* default configuration). The decrease at higher h_{BP} is due to an overall velocity decrease due to drastically enlarged cross section A (compare Eq. 2 and 3).

v_c is further increases with the applied overall flow rate, Q_{total}, (**c**). According to Eq. 2 and 9.1, both quantities are directly proportional.

g-l) Overall, both mixing time constants (Δt and τ) decrease with increasing size of the on-chip BP. The trends of τ (**j** and **k**) largely resembles those of Δt (**g** and **h**) as both quantities are interconnected.[1] In the on-site mixing configuration, both time constants decrease with decreasing w_{NC} and increasing h_{BP} due to the reduced diffusion length (w_{NC}) and increased convective flux in close proximity to the imaging area (IA), respectively. In the premixing configuration, an increase of both time constants is observed at intermediate w_{NC} which is

because the drop in convective transport inside the NC (see drop in flow velocity in **d**) is not (yet) compensated by the accelerated diffusive transport over too long diffusion lengths ($w_{NC} > 1.2$). The decrease with h_{BP} is rather abrupt, while the changes with w_{NC} are more gradual due to corresponding variation in relevant geometric parameters (BP cross-section and distance from the IA). Both for w_{NC} and h_{BP}, the improvements in the on-site mixing configuration (≈ 2 orders) exceed those for the premixing configuration (≈ 1 order) as latter is significantly accelerated by convective transport in the NC of the default scenario already.
Further, Δt decreases with the applied overall flow rate, Q_{total}, for both configurations operated with diffusion cells (**i**). This reflects the dependence of the travel time on Q_{total}. Over the entire range of Q_{total}, the absolute Δt-values remain larger for the premixing than for the on-site mixing configuration, reflecting the intrinsic delay caused by the extended premixing channel. In the premixing configuration, τ depicts a slight dependence on Q_{total}. The effect of the premixing channel can even be compensated with respect to the on-site mixing configuration. This is because solution replacement in the on-site mixing configuration is entirely dominated by diffusion, while convection is negligible (compare **e**).

Limitations of diffusion cell design

Diffusion cell prototypes were fabricated from standard E-chips (compare Methods section). The depth of the on-chip bypass is limited as the walls after reactive ion etching (RIE) are vertical (compare Supplementary Fig. 1a), while the grove forming the window from the back side is KOH-etched and thus has 45º inclination. This limitation can be overcome when using KOH etching also for the on-chip bypass channel (compare Supplementary Fig. 1b).
It must be noted that the applied manufacturing routine results in bare silicon (plus a native oxide layer) being exposed to the sample solution, which may influence on the stability and therefore eventually substances compatible to the chip. Nevertheless, these issues are easy to overcome in an optimized fabrication process, which however exceeds the scope of this work.

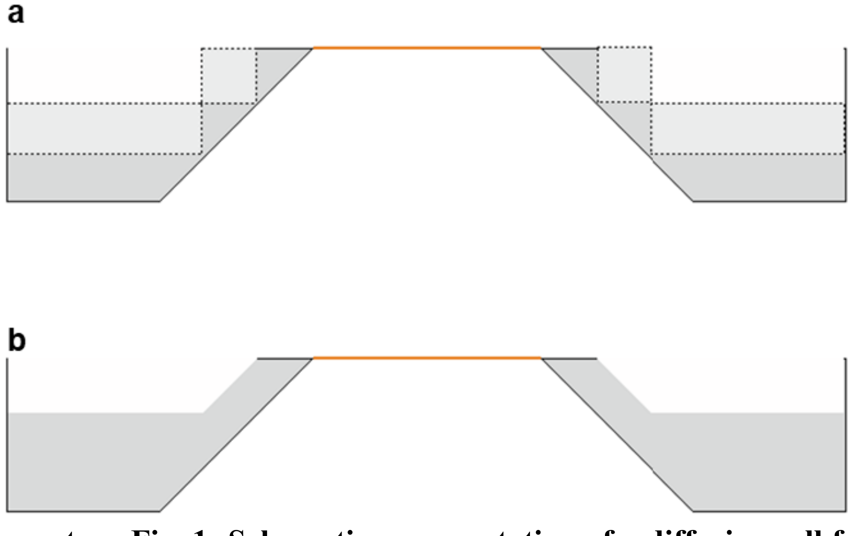

Supplementary Fig. 1: Schematic representation of a diffusion cell from different fabrication methods. Vertical walls from RIE limit the depth of the on-chip bypass channels (**a**), while inclined walls from KOH etching would overcome this limitation (**b**). In **a** and **b,** the SiN window is depicted in orange and the Si chip is represented in grey; continuous black lines represent the contour of unmodified chips. Dashed black lines in **a** illustrate that due to the inclined backside, the width of the central nanochannel is limited by the depth of the on-chip bypass channel, or vice versa, when RIE etching methods are applied.

Supplementary Note 3. Mass transport mechanisms in diffusion cell setups

Convective *vs.* diffusive transport

To evaluate the dominant mass transport in developed diffusion cell setups, convective and diffusive flux was analyzed in the 3D convection diffusion models. A series of parameters comprising volumetric flow rate, diffusion coefficient and flow channel configuration were screened. The corresponding data is depicted in Supplementary Fig. 2 – 4. It is crucial to realize the conceptual difference between net convective and diffusive flux of a solute: While convective flux (red curves below) enables continuous renewal (and/or removal) as long as the flow is switched on; net diffusive flux only arises when a concentration gradient is present, thus determining the mixing rate during solution replacement in microfluidic diffusion cells.
Further, the effect of diffusion on (initially sharp) concentration interfaces was evaluated to estimate diffusive broadening of an inflowing solution when restarting the flow at inactive inlets (Supplementary Fig. 5).

<u>Dependence on flow rate – direct flow with premixing configuration</u>

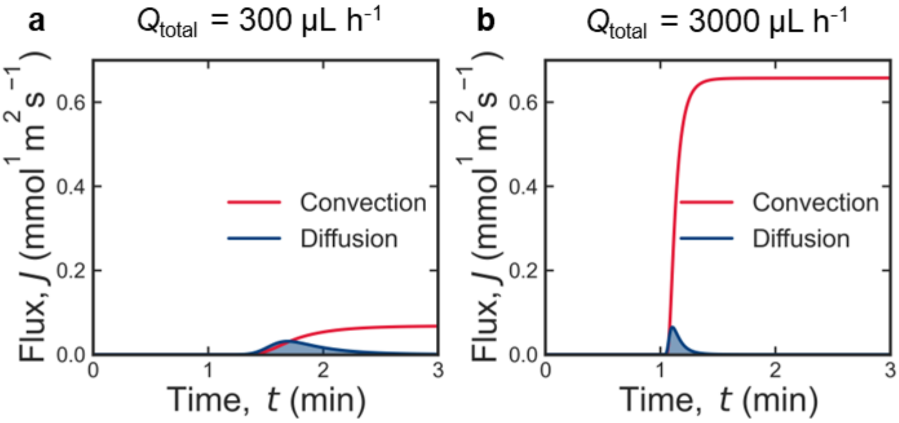

Supplementary Fig. 2: Contribution of convective and diffusive flux to solution replacement dynamics in direct flow with premixing configuration with diffusion cells (w_{NC} = 0.2 mm) at different flow rates. Contribution of convective (red line) and diffusive (blue) flux are depicted for flow rates of $Q_{total} = 300\ \mu L\ h^{-1}$ (**a**) and $Q_{total} = 3000\ \mu L\ h^{-1}$ (**b**). With increasing flow rate, the relevance of convective flux increases. The diffusion coefficient was $D = 10^{-10}\ m^2\ s^{-1}$. Note the conceptual difference between convective (continuous) and diffusive (depending on concentration gradients) flux.

Dependence on diffusion coefficient – direct flow with premixing configuration

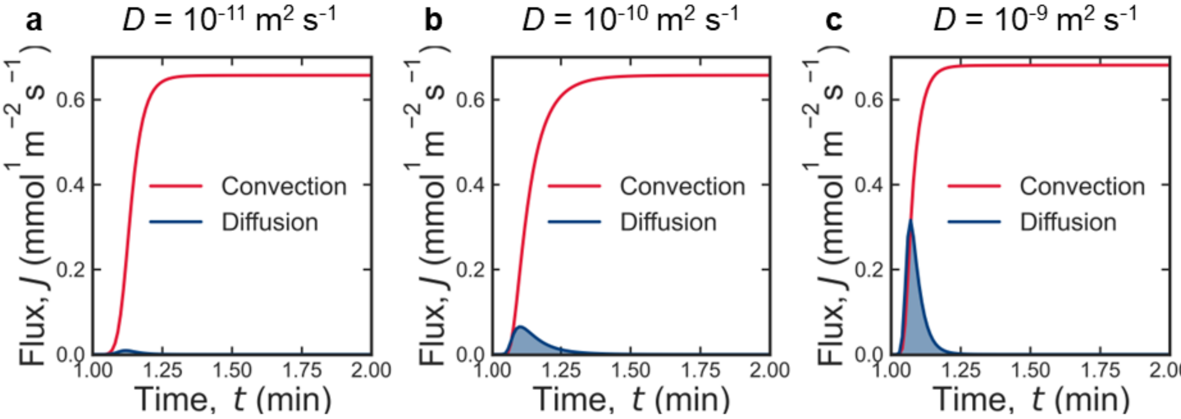

Supplementary Fig. 3: Contribution of convective and diffusive flux to solution replacement dynamics in premixing configuration with diffusion cells (w_{NC}=0.2 mm) at different diffusion coefficients. Contribution of convective (red curve) and diffusive (blue curve) flux to the solution replacement in the premixing configuration at $D = 10^{-11}$ m^2 s^{-1} (**a**), $D = 10^{-10}$ m^2 s^{-1} (**b**) and $D = 10^{-9}$ m^2 s^{-1} (**c**). Relevance of diffusive flux increases with increasing D. Flow rate was $Q_{total} = 3000$ µL h^{-1}.

Dependence on diffusion coefficient – bathtub with on-site mixing configuration

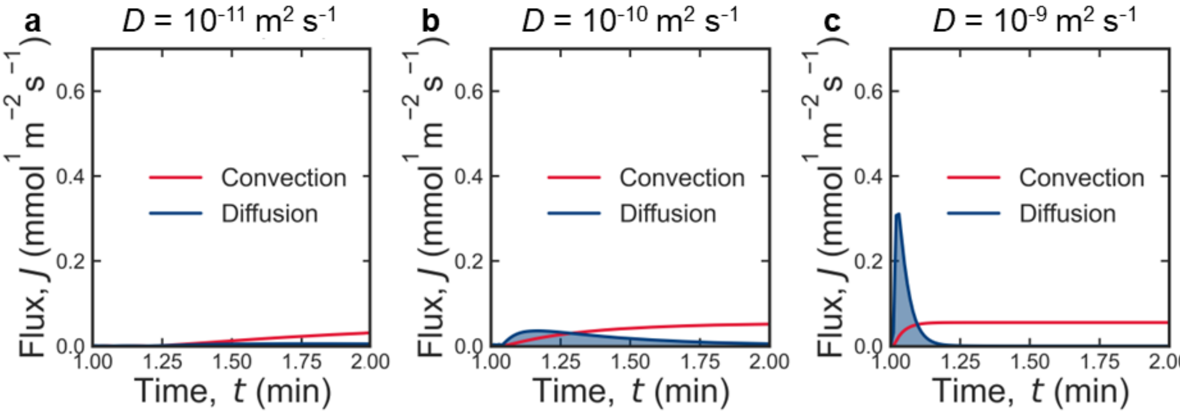

Supplementary Fig. 4: Contribution of convective and diffusive flux to solution replacement dynamics in bathtub with on-site mixing configuration with diffusion cells (w_{NC} = 0.2 mm) at different diffusion coefficients. Contribution of convective (red curve) and diffusive (blue curve) flux to the solution replacement in the bathtub with on-site mixing configuration at $D = 10^{-11}$ m^2 s^{-1} (**a**), $D = 10^{-10}$ m^2 s^{-1} (**b**) and $D = 10^{-9}$ m^2 s^{-1} (**c**). Diffusive flux has significant effect on solution replacement for a wide range of D. Flow rate was $Q_{total} = 3000$ µL h^{-1}.

Diffusive broadening – zero flow

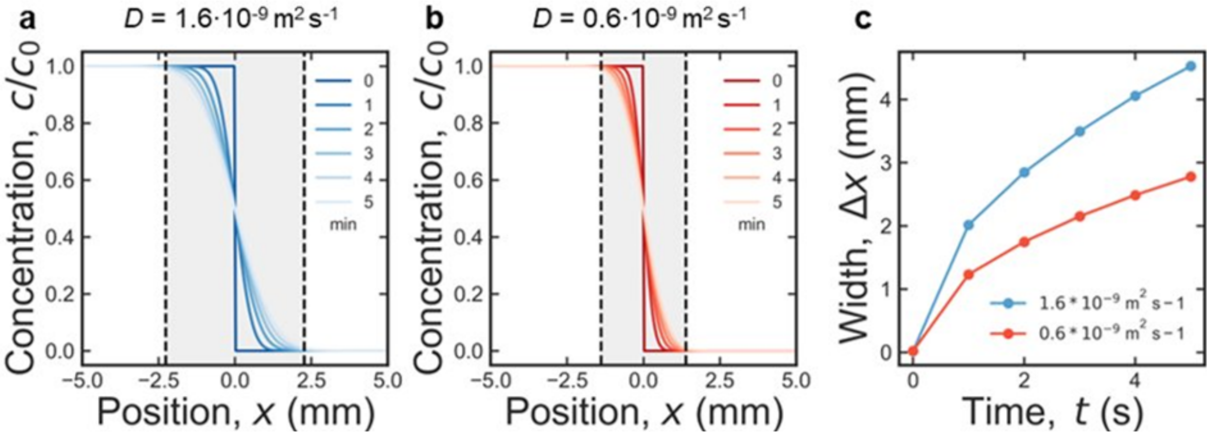

Supplementary Fig. 5: Diffusive broadening of sharp concentration interfaces (zero flow). a, b Concentration profiles along initially sharp solute interface with increasing time (up to 5 min) for two diffusion coefficients, i.e., $1.6 \cdot 10^{-9}$ and $0.6 \cdot 10^{-9}$ m^2 s^{-1}.[1,3] **c** Width, Δx, of the concentration gradient upon diffusive broadening with time t. Δx at t = 5 min is indicated by grey background and vertical dashed lines in **a** and **b**, respectively. The broadening affects the inflow concentration gradient when initially stopped inlets are switched on, e.g., in a solution replacement experiment. Refer to Supplementary Fig. 10 for effect on time constants.

Effect of window bulging

The effect of window bulging on the hydrodynamic properties of diffusion cell setups was extensively studied both experimentally and by numerical simulations. Both experiments and numerical modelling in realistic 3D geometry revealed a negligible variation of replacement dynamics across the IA (Supplementary Fig. 6, 7 and 9). Numerical modelling further provided more profound insights: the 3D models enabled estimating the effect of bulging on characteristic time constants of solution replacement, and compare it to other aspects (e.g., diffusive broadening; Supplementary Fig. 10). Additionally, simplified diffusion models provided profound understanding of the effect of bulging on solution replacement dynamics (see Supplementary Note 3).

Experiments

Concentration curves across IA

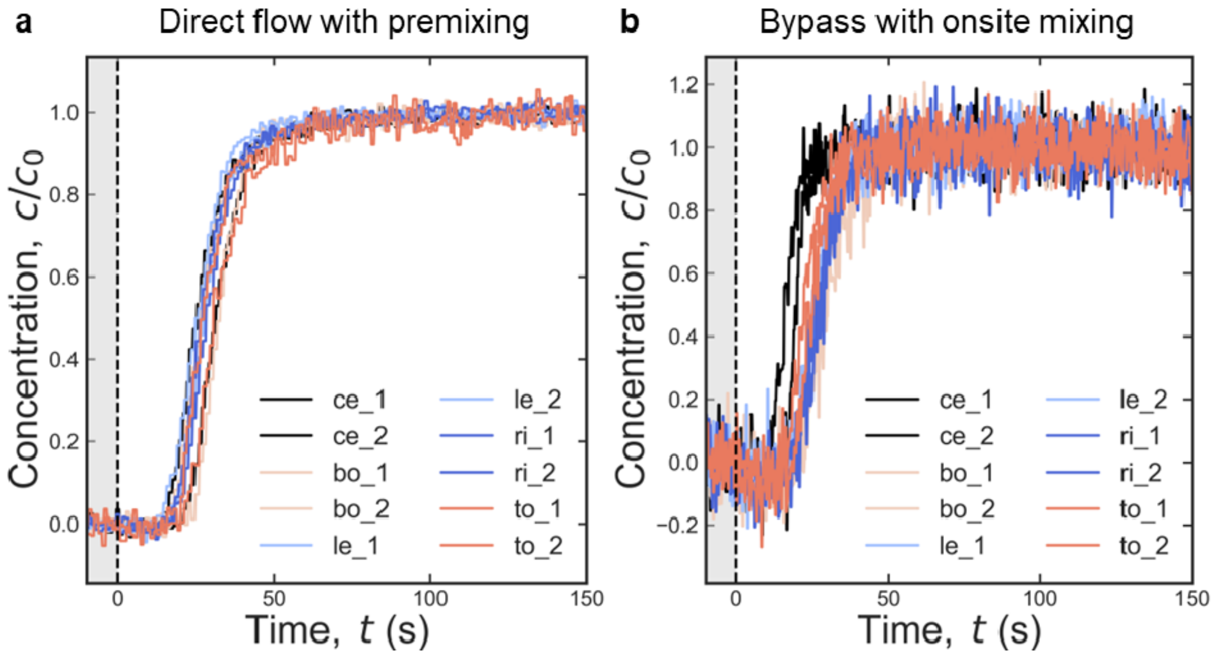

Supplementary Fig. 6: Experimental concentration curves across IA. Several normalized concentration curves extracted at the center (ce) and 4 edges, i.e., top (to), bottom (bo), left (le) and right (ri), of the IA (nominal 20 × 20 μm^2) for a diffusion cell operated in direct flow with premixing (**a**) and bypass with on-site mixing (**b**) setup, respectively. Note the nearly identical solution replacement dynamics across the entire IA. Supplementary Fig. 7 depicts extracted time constants. Vertical dashed black line indicates the timepoint at which the active inlet switches from reference (pure water, grey background) to contrast agent solution (PTA, white background).

Experimental time constants across IA

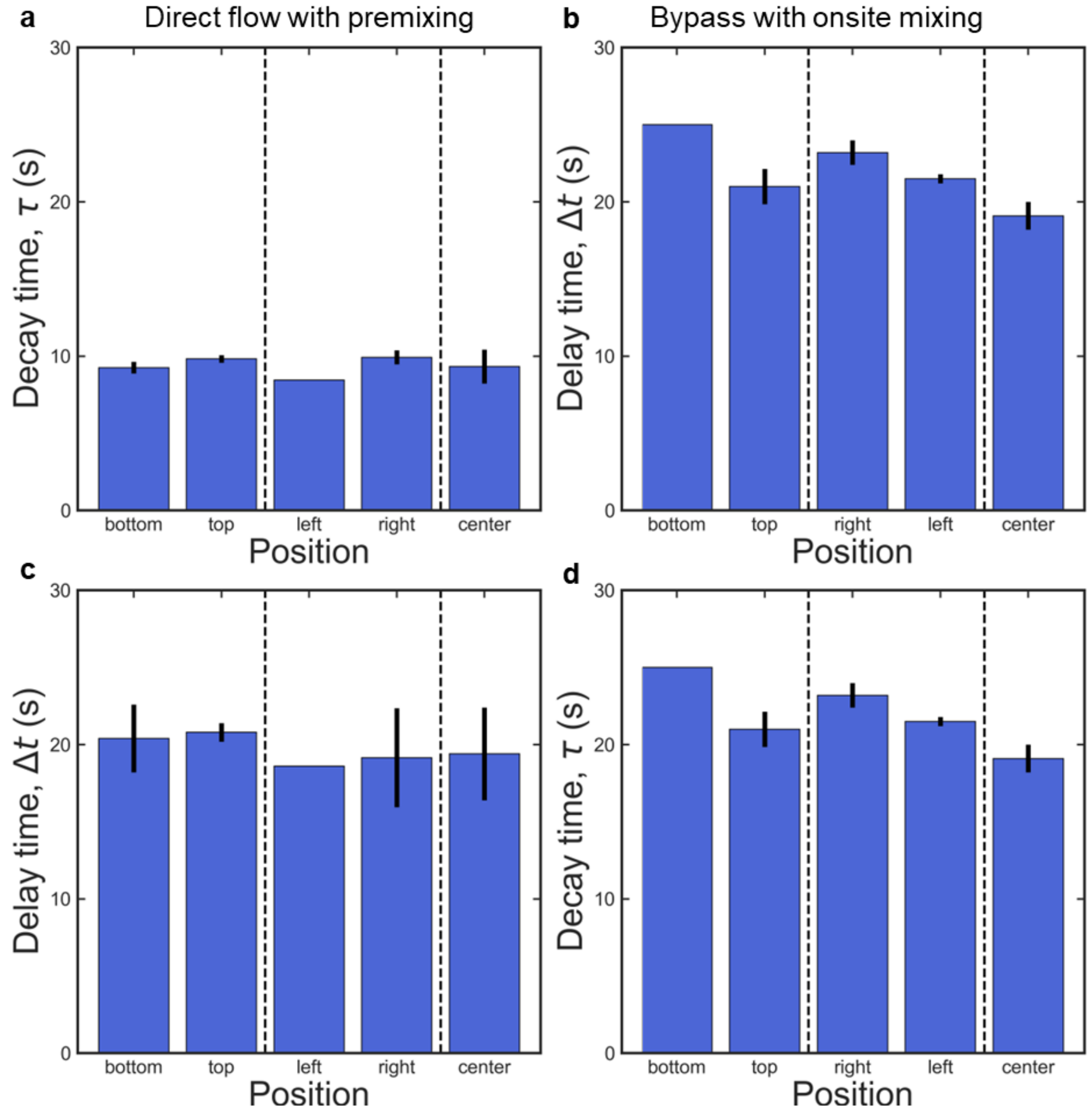

Supplementary Fig. 7: Experimental mixing time constants across the IA. Delay (**a, c**) and decay (**b**, **d**) time constants at the bottom, top, left and right edge of the viewing area as well as in its center obtained when operating a diffusion cell in the direct flow with premixing and bypass with on-site mixing setups, respectively. The variation across the viewing area is below the accuracy of the image contrast variation method (manly determined by the manual synchronization of flow control and imaging, see error bars). Time constants were extracted from data depicted in Supplementary Fig. 6, i.e. error bars represent standard deviation for two independent measurements.

Simulations – realistic 3D convection diffusion models

Implementation of bulging

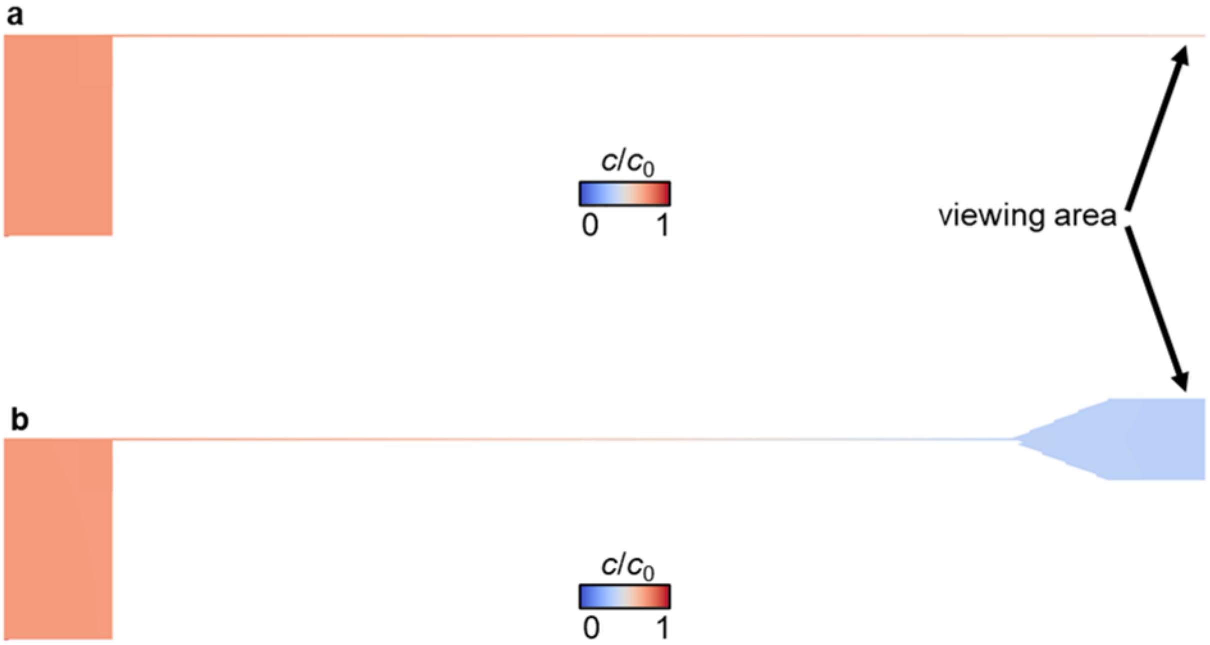

Supplementary Fig. 8: The effect of window bulging on solution replacement in the central nanochannel. a, b 2D concentration profile of solute in on-site mixing configuration neglecting (**a**) and considering (**b**) window bulging at 5 s after applying contrast agent flow (w_{NC} = 0.2 mm, Q_{total} = 1200 µL h^{-1}). Bulging in **b** was defined based on information provided by the manufacturer[4] and was qualitatively approximated with truncated pyramids (width: w = 20 µm; height: h = 2 µm) for meshing purposes. Aqueous solution in the additional volume is replaced predominantly via diffusion resulting in a delayed and more gradual replacement in the viewing area. Refer to Supplementary Fig. 9 and 10 for effect on time constants.

Simulated concentration curves across IA

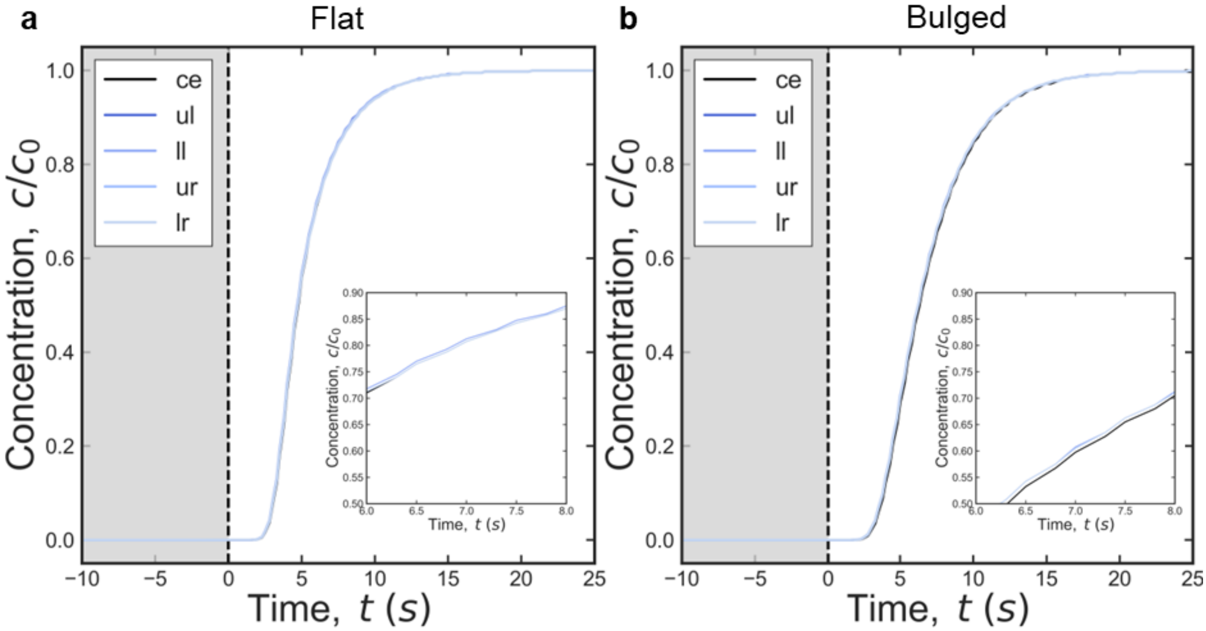

Supplementary Fig. 9: Simulated concentration curves across IA. Normalized concentration curves extracted at the center and 4 corners (upper left (ul), lower left (ll), upper right (ur), lower right (lr)) of the viewing area (20 × 20 μm^2) in a model representing a diffusion cell operated in bypass with on-site mixing setup with flat (**a**) and bulged (**b**) IA. Solution replacement is homogeneous across the entire IA. Refer to Supplementary Fig. 8 for details on implementation of bulging. The dashed black lines indicate the timepoint at which the active inlet switches from reference (pure water, grey background) to contrast agent solution (PTA, white background).

Effect of bulging on time constants

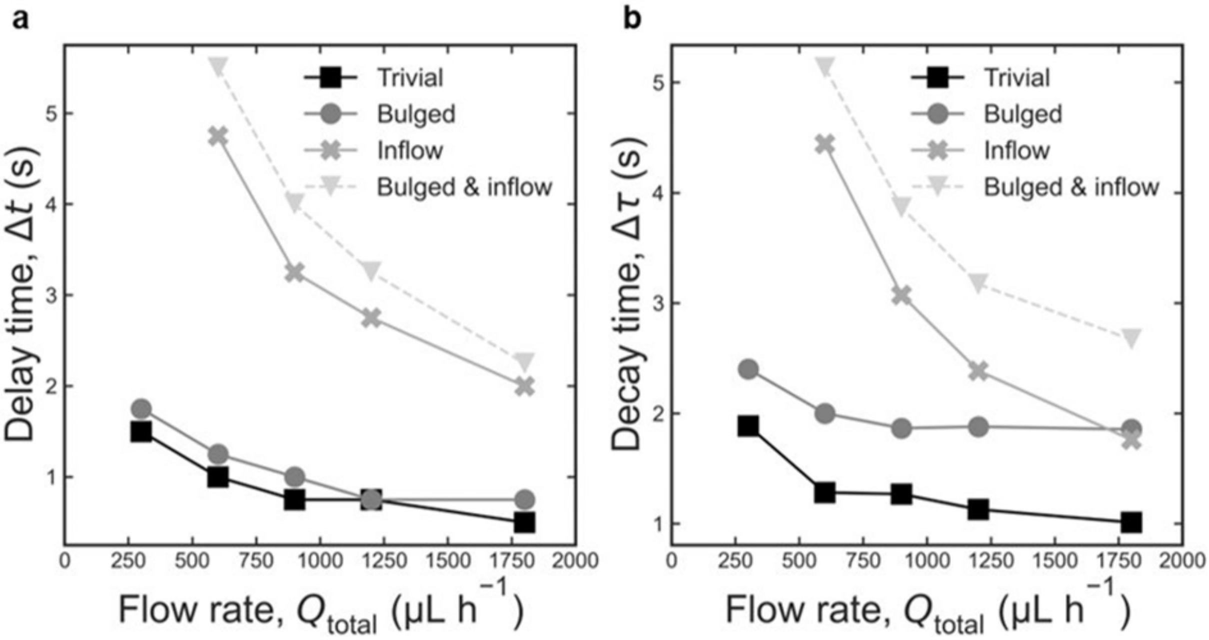

Supplementary Fig. 10: Refinement of simulated time constant of solution replacement in bathtub with on-site mixing configuration with diffusion cells (compare Fig. 4). Flow rate dependence of delay time (**a**) and decay time constant (**b**) for different models. The black rectangles correspond to the non-refined trivial model which considers geometry of the diffusion cell predicting too rapid solution replacement dynamics. More sophisticated models estimate window bulging (dark grey circles), inflow gradients (grey crosses); note the rather low/strong dependence of window bulging and inflow gradients on flow rate, respectively. The combination of both effects shows accurate agreement with experimental values (light grey triangles) as depicted in Fig. 4f-i (main manuscript).

Simulations – 2D axisymmetric diffusion model

A geometrically simplified model (2D axisymmetric) of the central nanochannel was implemented to evaluate the effect of bulging on solution replacement dynamics in detail. Supplementary Fig. 11 is a schematic representation of the model geometry; Supplementary Fig. 12 and 13 compare simulated concentration curves and diffusive fluxes in flat and bulged model geometries, respectively.

Implementation of bulging in simplified model geometry

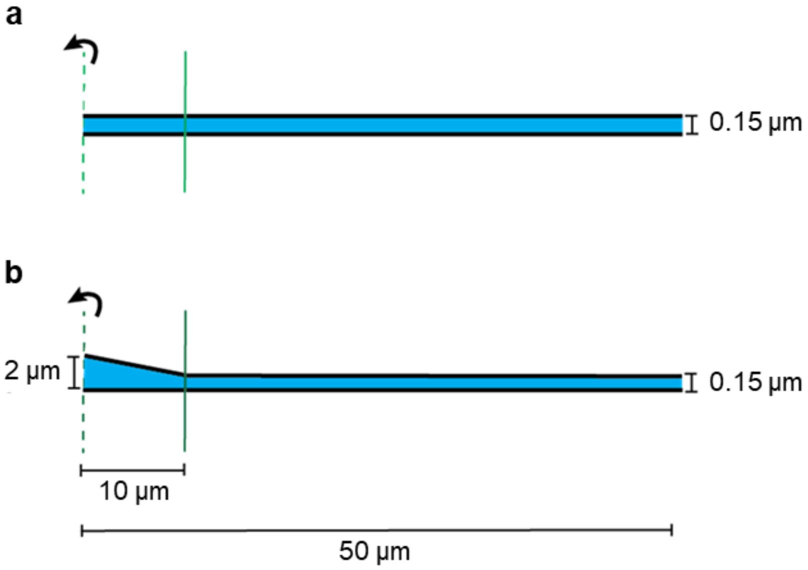

Supplementary Fig. 11: Schematic representation of different 2D axisymmetric diffusion models. a, b Entirely flat (**a**) and partially bulged (**b**) nanochannel geometries were implemented. Bulged segment was represented by triangular cross-section (9.25 µm^2) representing 20 µm wide window area (radius: 10 µm) and 2 µm of expansion in the center. Note that the bulging parameter serve as a good first approximation.[4] In both scenarios the radius of the nanochannel was 50 µm. Dashed lines represent the rotational center of the 2D axisymmetric model; continuous lines are located at 10 µm, i.e., at the onset of bulging in **b**. Note that bulged and flat area in **b** are not entirely depicted to scale for better visibility.

Simulated concentration curves across IA

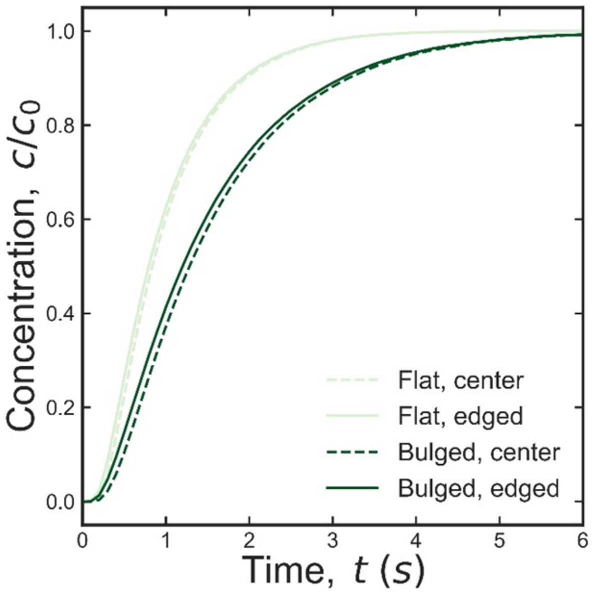

Supplementary Fig. 12: Concentration curves of solution replacement at different positions in flat and bulged model geometries. Concentration curves were extracted in the center (r = 0) and at r = 10 um (edge ≙ transition from the bulged to the flat channel section). For the model geometry (chosen arbitrarily within representative regime; compare Supplementary Fig. 11),[4] the effect of bulging is more pronounced than the dependence on position across the IA.

Effect of bulging on diffusive flux

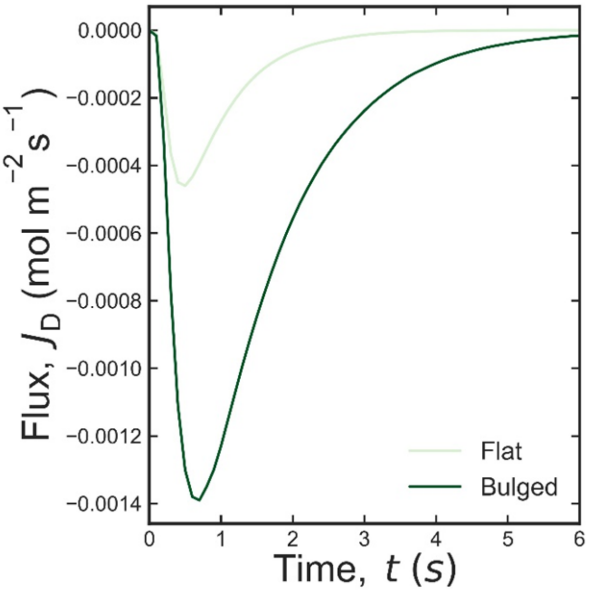

Supplementary Fig. 13: Radial diffusive flux (J_D) at the transition from the flat to the bulged section of the nanochannel. Negative radial diffusive fluxes indicate inward diffusion. The absolute values are larger for the bulged setup compared to the entirely flat setup. Thus, despite the lower concentration values detected in Supplementary Fig. 12 diffusive transport is increased due to bulging, i.e., more solute enters the IA within a given time.

Supplementary Note 4. Physical prototyping of ex-situ sample holder

The development of LP-TEM sample holder and the experimental quantification through electron microscopical methods are expensive and time-consuming. Due to economic constraints, both regarding fabrication and further experimental verification we developed ex-situ prototyping routines comprising ex-situ prototypes and optical means for their characterization.

Fabrication of ex-situ sample holders

An ex-situ prototype of a direct flow with on-site mixing setup was purchased from external collaborators (Mondragon University) upon specific request. The internal geometry of the manufactured prototype was derived from the Poseidon sample holder series of Protochips company.[2] Hence, the channel geometry in the bathtub region was identical to that of the Poseidon Select sample holder (computer-aided design (CAD) files were obtained from Protochips Inc. under non-disclosure agreement, NDA); however, no premixing channel is present to supply liquids. Instead, external channels are connected directly to three individual pockets (with the two inlets opposing each other) as characteristic for the Poseidon 200 setup.[1] The external features of the ex-situ prototype were designed to fit those of standard UV-vis cuvettes ($1 \times 1 \times 4$ cm^3). The frontside view of the ex-situ prototype is depicted in Supplementary Fig. 16b. In- and outlet tubings were connected directly from the backside.

Optical image contrast variation method

The image contrast variation method (established for TEM previously[1]) was adapted for UV-vis spectroscopy to quantify solution replacement dynamics of LP-TEM flow reactor prototypes. Optical methods are widely applied for the hydrodynamic characterization and quantification of microfluidic devices where typical channel thicknesses of several hundreds of micrometres allow to operate at low optical contrast agent (dye) concentrations leading to satisfying (image) contrast variations.[5] The main challenge of adapting the spectroscopic approach to LP-TEM systems was in the miniaturization of central nanochannel, and consequently identifying contrast agents that induce measurable contrast variations given average nominal liquid thickness of ≤ 1 µm. Potassium permanganate ($KMnO_4$, $\varepsilon_{max} = 2.66 \cdot 10^4$ M^{-1} cm^{-1})[3] was identified as suitable candidate; saturated $KMnO_4$ solutions of ≈ 7 mM induced $\approx 8\%$ variation of the measured transmitted intensity at maximum extinction wavelength, $\lambda_{max} = 546$ nm. The strong oxidizing character of the permanganate and the risk of MnO_2 precipitation, should be noted to possibly limit its applicability for hydrodynamic characterization of LP-TEM reactors.[6,7] Details on the ex-situ and in-situ concentration calibration by optical means are depicted in Supplementary Fig. 14 and 15, respectively.

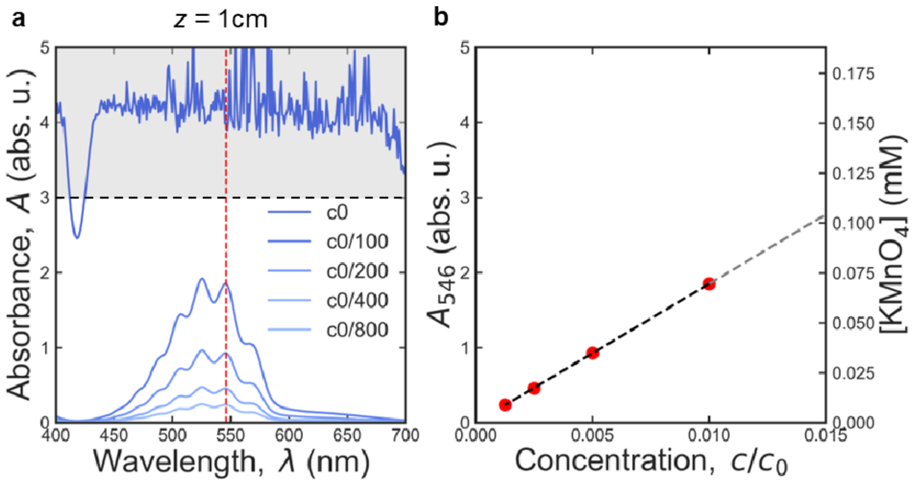

Supplementary Fig. 14: Concentration calibration of $KMnO_4$ solution in ex-situ UV-vis measurements (optical path $z = 1$ cm). a Absorbance, A, of $KMnO_4$ solution in the wavelength range $400 < \lambda < 700$ nm for various concentrations, c. c_0 denotes the concentration of the concentrated solution used for hydrodynamic calibration described in the main manuscript; its optical signal exceeds the detection limit (grey area) in classical ex-situ experiments with cuvettes of 1 cm. Dashed black line indicates detection limit of the spectrometer. **b** Absorbance, A_{546}, extracted at wavelength of the maximum ($\lambda_{max} = 546$ nm; red dashed line in **a** from the curves in **a**. The measured data points fall into the linear regime of Beer's law; linear extrapolation assuming $\varepsilon_{KMnO4,546\ nm} = 2.66 \cdot 10^4\ M^{-1}\ cm^{-1}$ for the extinction coefficient of $KMnO_4$ at 546 nm allows to estimate $c_0 \approx 7$ mM.[3]

Data post-processing

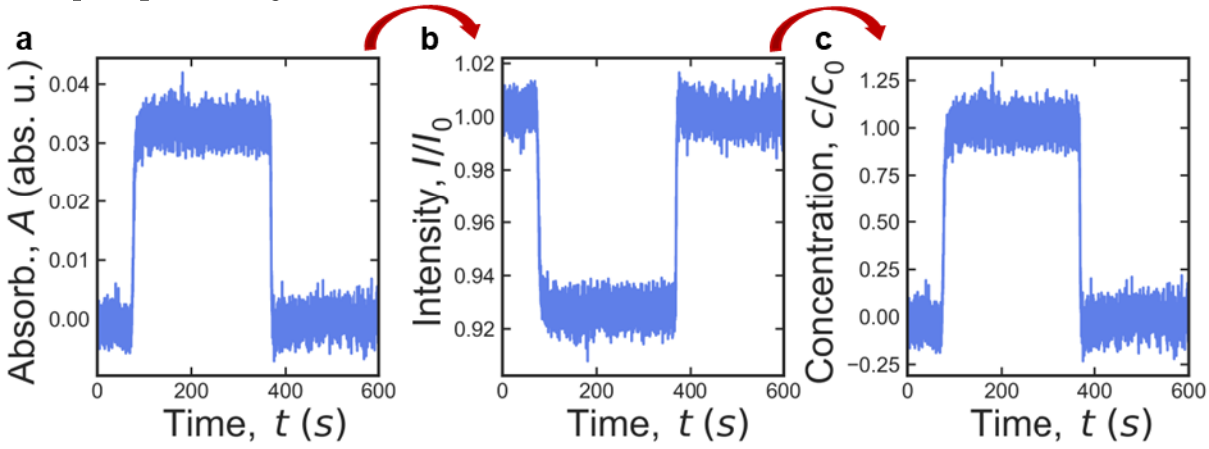

Supplementary Fig. 15: Post-processing routine for hydrodynamic calibration via UV-vis spectroscopy. The absorbance, A, (**a**) is measured across the entire viewing area; it is converted in normalized intensity, I/I_0 (**b**); which is finally expressed as normalized concentration, c/c_0, (**c**) following concentration calibration routines.[1]

Hydrodynamic characterization

To quantify the hydrodynamic properties of diffusion cells operated in the direct flow with on-site mixing ex-situ prototype, a spectrometer was used to acquire time-dependent concentration profiles (see Supplementary Fig. 16c) by tracking changes of the measured absorbance, A, (see

Methods section for conversion into transmitted intensity and solute concentration) at 10 Hz across the entire viewing area (≈ 20 × 20 µm^2). The solute-induced signal variation was well above noise level and reproducible, yet fluctuations were more pronounced than in TEM data. Characteristic time constants were extracted to quantify improved replacement dynamics. Quantitative analysis (Supplementary Fig. 16d and e) shows that the delay time and decay time constant were Δt = 12 s and τ = 1.8 s (corresponding to 90% of solution being replaced in ≈ 4 s; Q_{total} = 2400 µL h^{-1}), respectively. This acceleration with respect to data depicted in Fig. 4 can only be partially ascribed to geometrical improvements given the faster diffusion of the optical dye $D_{MnO4-} \approx 1.6 \cdot 10^{-9}$ m^2 s^{-1} > D_{PTA}.[1,3] The experimental constants of solution replacement, particularly τ, are accurately reproduced with a refined numeric model which estimates window bulging and adjusted gradual inflow gradients (grey triangles in Supplementary Fig. 16d and e; compare Supplementary Note 4).

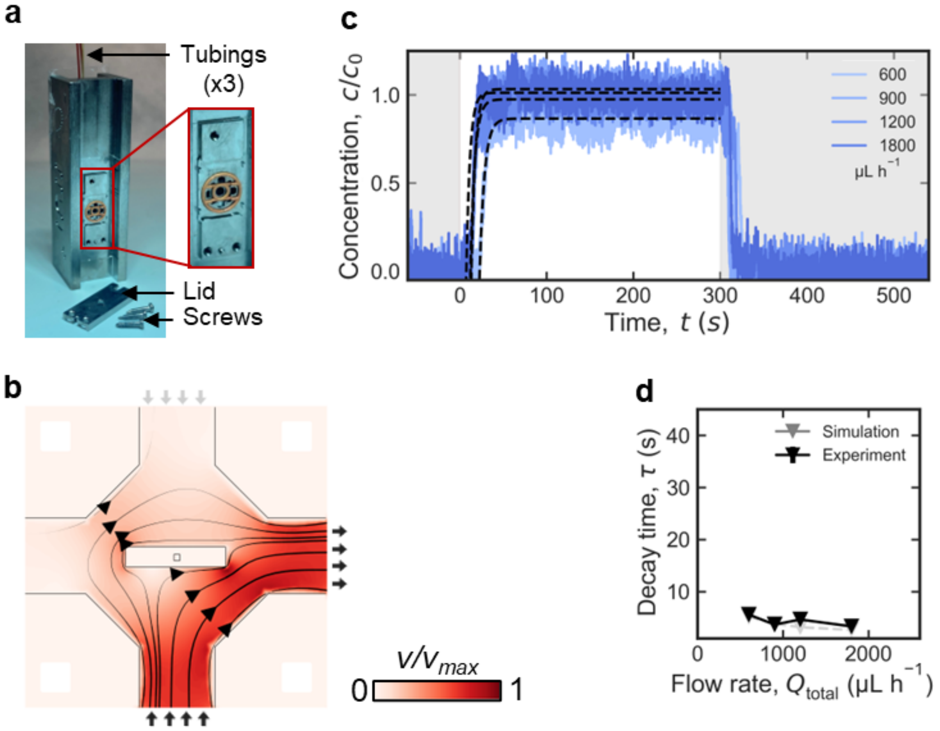

Supplementary Fig. 16: Hydrodynamic quantification of direct flow with on-site mixing LP-TEM flow setup with diffusion cells. a Prototype of a direct flow with on-site mixing configuration constructed on the basis of Protochips' Poseidon holders. The system relies on gasket technology for sealing; no premixing channel is present as tubings are directly connected to individual pockets from behind. **b** Illustrative representation of the flow profile in the diffusion cell. Note that the velocity is increased compared to the bathtub with on-site mixing setup due to reduced off-chip bypass. Refer to main manuscript for details. **c, d** The system was used for ex-situ flow calibration via UV-vis spectroscopy. Example curves of normalized concentration (blue) for different flow rates, Q (**c**) tracked across the entire IA using potassium permanganate ($KMnO_4$; $D_{MnO4-} \approx 1.6 \cdot 10^{-9}$ m^2 s^{-1}) as optical contrast agent.[3] Black dashed lines in **c** represent exponential fits. Experimental decay time constants, τ, (**d**) of solution replacement (black triangles) were reproduced by numeric modelling (grey).

Supplementary Note 5. Further scenarios and improved experimental methods

Direct flow with on-site mixing setup derived from Poseidon 200 sample holder

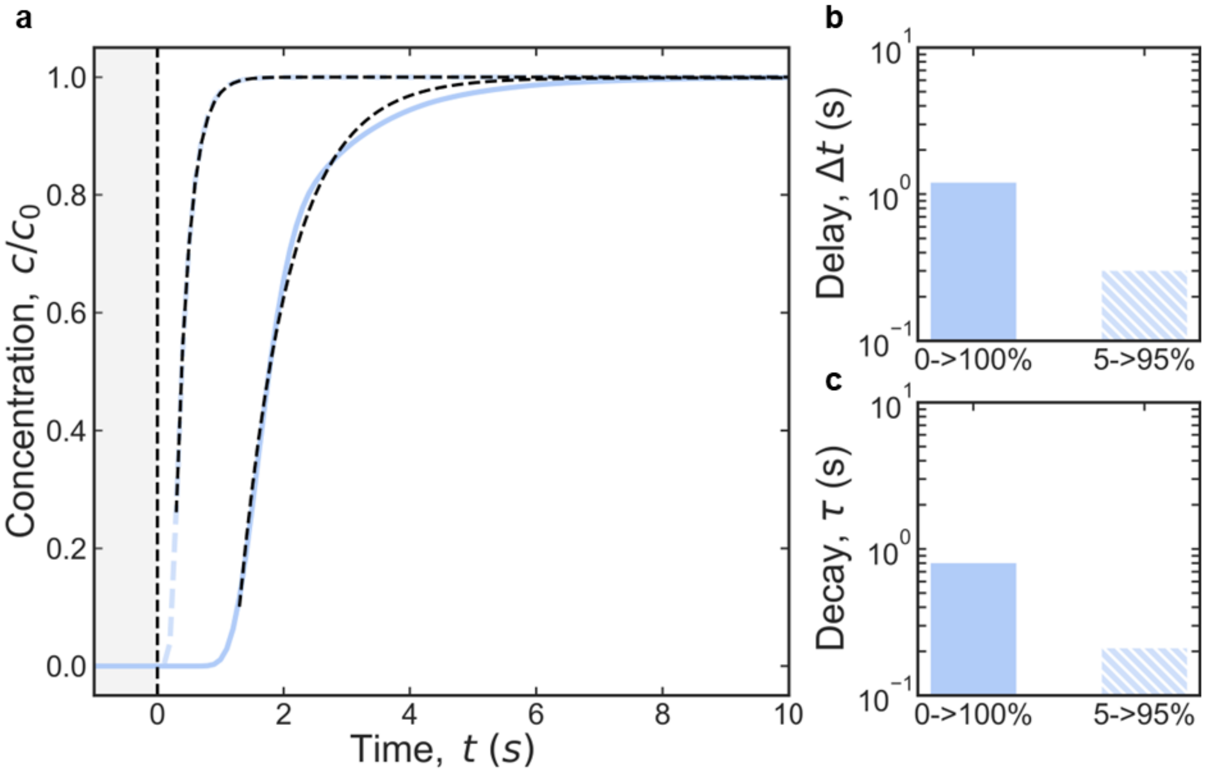

Supplementary Fig. 17: Solution replacement in direct flow with on-site mixing configuration under optimized experimental conditions. **a** Simulated time-dependent concentration profile at the center of the nanochannel induced through conventional (Q_{CA}: 0 → 1·Q_{total}) and optimized (Q_{CA}: 0.05·Q_{total} → 0.95·Q_{total}; reproduced from Fig. 5c) experimental methodology. Characteristic parameters were: w_{NC} = 0.05 mm, l_{NC} = 0.65 mm, h_{NC} = 150 nm, h_{BP} = 50 µm. Dashed black curves represent exponential fits; vertical dashed black line indicates the timepoint at which the active inlet switches from reference (pure water, grey background) to contrast agent solution (PTA, white background). **b**, **c** Extracted time constants of solution replacement, Δt (**b**) and τ (**c**), for the curves in **a**. Preventing mixing at the channel entrance results in significantly (> 1 order of magnitude) improved time constants leading the way to sub-second solution replacement dynamics. The diffusion coefficient was $D = 1.3\cdot10^{-9}$ m^2 s^{-1} to reflect more realistic scenarios of diffusing small molecules.[8]

Virtual double-inlet diffusion cell for DENSsolutions' stream system

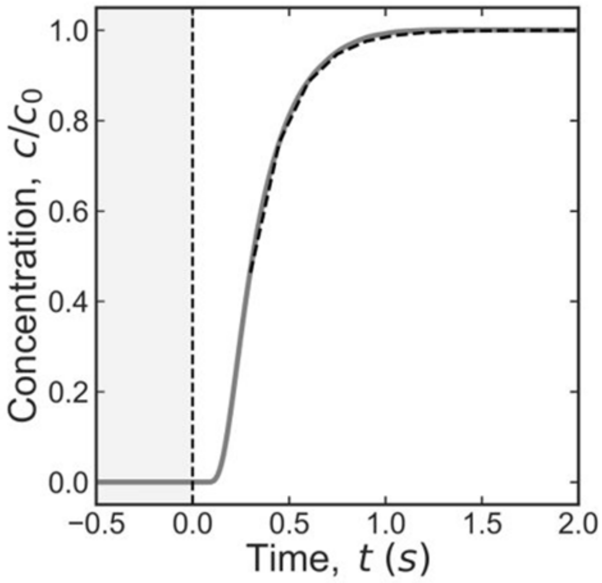

Supplementary Fig. 18: Solution replacement in virtual double-inlet diffusion cell compatible with DENSsolutions Stream system. Simulated time-dependent concentration profile at the IA. The geometrical parameters were h_{BP} = 50 µm, w_{channel} = 1 mm, w_{NC} = 50µm, Q_{total} = 3000 µL h^{-1} (resulting in $\Delta p \approx 25$ mbar and $v_c = 3.5 \cdot 10^{-7}$ m s^{-1}) and $D = 1.3 \cdot 10^{-9}$ m^2 s^{-1}.[8] Dashed black lines represent exponential fits. Delay time and decay time constants were determined Δt = 0.15 s and τ = 0.19 s.

Supplementary Note 6. Application experiment

Agarose film fabrication

Agarose thin-films of controlled thickness were obtained via spin-coating routines (see Methods section).

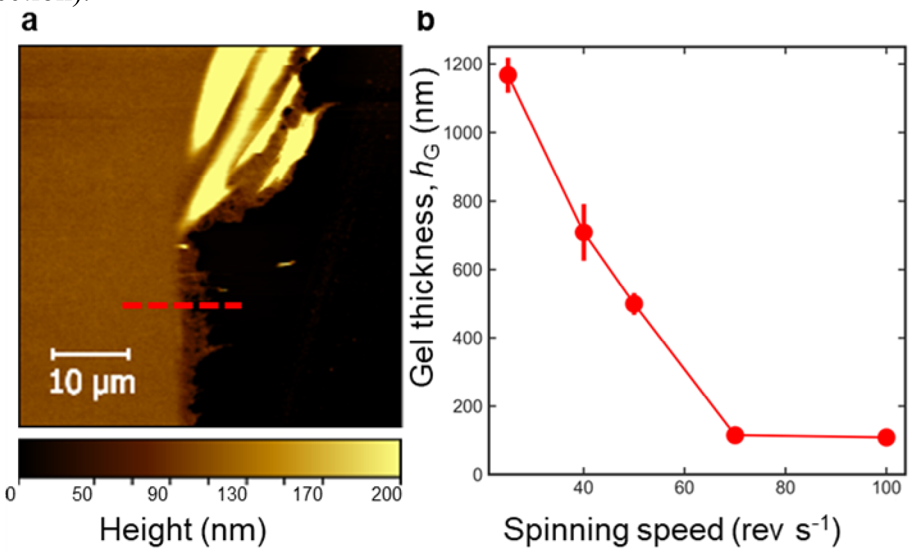

Supplementary Fig. 19: Preparation of agarose thin-films via spin coating. a Representative topographic AFM image of a spin-coated thin film of agarose gel on SiN membrane under 100% humidity. Homogeneous gel layers formed (left side in **a**); their height was measured across scratches (right side in **a**) as illustrated by red dashed line. **b** Dependence of the gel thickness z on the spinning speed (revolutions per second, rev s^{-1}; duration: 15 s) measured across scratches. 70 rev s^{-1} were selected to prepare thin-film fitting LC assemblies with 150 nm spacer thickness (indicated by black arrow in **b**). Error bars represent standard deviation of four independent measurements.

Triggering of nanoscale dynamics through rapid solution replacement

The capability of rapid solution replacement to replicate mixing condition in-situ was demonstrated by altering beam-mediated dynamics of confined AuNPs (refer to Methods section of the main document for experimental details). Supplementary Fig. 19a exemplarily depicts an image of the acquired sequences, which was overlayed with the paths of 12 particles present in the IA tracked in ImageJ software. The displacement Δx of these particles *vs.* time is depicted in Supplementary Fig. 19b. Two mobility regimes are clearly distinguishable: while in pure water (white background in **b**), the mobility is strongly suppressed ($0 < \Delta x < 5$ nm), in NaCl solution (c = 100 mM; grey) particles could hop distances of several tens of nanometers.

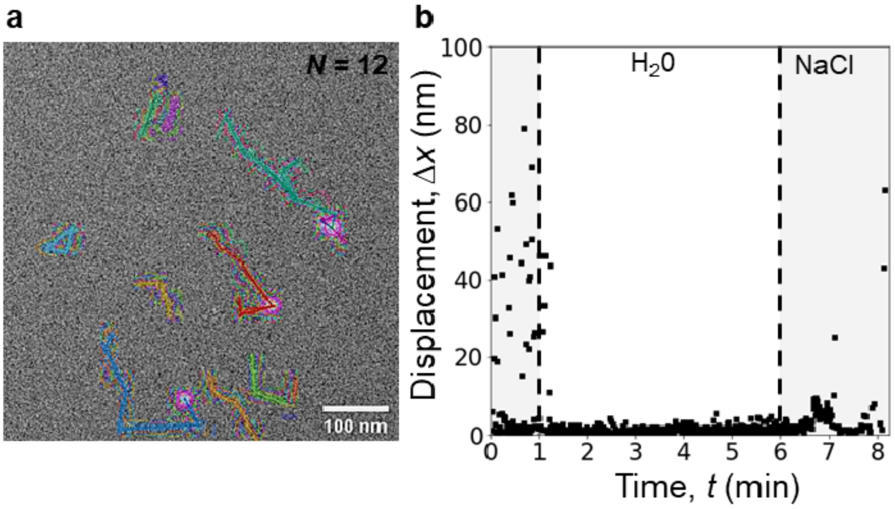

Supplementary Fig. 20: Inducing nanoscale dynamics through rapid solution replacement. a Representative LP-TEM image of AuNPs in agarose gel (nominal thickness: 150 nm; compare black arrow in Supplementary Fig. 19b) extracted from Supporting Movie 1. The position of particles in the IA ($N = 12$) was tracked during 8 min of experiment (coloured lines overlayed to the image). The color scale is inverted to improve traceability of the particles. **b** Time-dependent displacement (Δx) of all tracked particles during the solution replacement experiment depicted in **a**. Two mobility regimes are apparent: grey and white background indicate active water and NaCl solution ($c = 100$ mM) flow ($Q_{total} = 500$ µL h^{-1}).

While the hopping indicates increased particle motion due to high ionic strength,[9] the non-zero displacement in pure water environment might indicate beam-induced damage of the agarose gel matrix. The latter hypothesis is supported by the rectilinear motion of various particles during elongated time periods as illustrated by blue, orange, red and turquoise tracks in Supplementary Fig. 20a. In fact, radiolytic damage of bio-polymeric composite materials has been widely observed in similar scenarios.[10,11]

More relevant in the context of this work, however, are the dynamic aspects of the transition between both mobility regimes since the delay after which transitions occur reflect the solution exchange dynamics. Supplementary Fig. 20d reveals time delay as short as few tens of seconds. Accounting for hindrance of diffusion in agarose gel,[12] these values are in good agreement with calibrated time constants reported in the main manuscript. Comparable experiments with unmodified LC setup appeared to be nearly impossible due to tremendously increased time constants.

To our knowledge, the displayed experiment thus represents the first ever example of mixing times in the regime of seconds observed on a sample deposited prior to the assembly of the LP-TEM flow reactor. The achieved mixing times are in the range of standard ex-situ setups and therefore exciting for correlative studies. Optimizing experimental and imaging conditions are required for more quantitative understanding on the investigated process.

Supplementary References

1. Merkens, S. *et al.* Quantification of reagent mixing in liquid flow cells for Liquid Phase-TEM. *Ultramicroscopy* **245**, 113654 (2023).

2. Protochips Inc. Protochips. *https://www.protochips.com/* https://www.protochips.com/ (2023).

3. Zenita Devi, O., Basavaiah, K. and Vinay, K. B. Application of Potassium Permanganate to spectrophotometric assay of metclopramide hydrochloride in Pharmaceuticals. *J. Appl. Spectrosc.* **78**, 873–883 (2011).

4. Protochips Inc. *Protochips' Guide to Poseidon E-Chips Version 1.3*. (2018).

5. Vakili, M. *et al.* 3D Micromachined Polyimide Mixing Devices for in Situ X-ray Imaging of Solution-Based Block Copolymer Phase Transitions. *Langmuir* **35**, 10435–10445 (2019).

6. Swain, H. A., Lee, C. and Rozelle, R. B. Determination of the Solubility of Manganese Hydroxide and Manganese Dioxide at 25°C by Atomic Absorption Spectrometry. *Anal Chem* **47**, 1135–1137 (1975).

7. Cohen, J. U. and Oddy, R. W. The stability of permanganate solutions. *Journal of the Society of Chemical Industry* **9**, 17–18 (1890).

8. E. Samson, J. Marchand and A. Snyder. Calculation of ionic diffusion coefficient on the basis of migration test results. *Mat. Struct.* **36**, 156–165 (2003).

9. Woehl, T. J. and Prozorov, T. The Mechanisms for Nanoparticle Surface Diffusion and Chain Self-Assembly Determined from Real-Time Nanoscale Kinetics in Liquid. *Journal of Physical Chemistry C* **119**, 21261–21269 (2015).

10. Korpanty, J., Parent, L. R. and Gianneschi, N. C. Enhancing and Mitigating Radiolytic Damage to Soft Matter in Aqueous Phase Liquid-Cell Transmission Electron Microscopy in the Presence of Gold Nanoparticle Sensitizers or Isopropanol Scavengers. *Nano Lett* **21**, 1141–1149 (2021).

11. Parent, L. R., Gnanasekaran, K., Korpanty, J. and Gianneschi, N. C. 100th Anniversary of Macromolecular Science Viewpoint: Polymeric Materials by In Situ Liquid-Phase Transmission Electron Microscopy. *ACS Macro Lett* **10**, 14–38 (2021).

12. Johnson, E. M., Berk, D. A., Jain, R. K. and Deen, W. M. Hindered Diffusion in Agarose Gels: Test of Effective Medium Model. *Biophys J* **70**, 1017–1026 (1996).
