# Supplementary material for: Toward sub-second solution exchange dynamics in flow reactors for liquid-phase transmission electron microscopy
Source: Nat Commun. 2024 Mar 21;15:2522. doi: 10.1038/s41467-024-46842-3 (PMC10957994; doi:10.1038/s41467-024-46842-3)
Supplement: Supplementary file 3 — Description of Additional Supplementary Files [file 41467_2024_46842_MOESM3_ESM.pdf]

**Description of Additional Supplementary Files**

File Name: Supplementary Movie 1
Description: TEM movie displaying the interaction of AuNPs in an agarose gel matrix with the SiN window surface. The mobility of the nanoparticles is controlled by changing the NaCl concentration through connected syringes. The total flow rate was 500 $\mu L\ h^{-1}$. The magnification was 27000×, the dose rate was 5 $e^{-}\ nm^{-2}\ s^{-1}$ and the image acquisition time was 1 s. The movie depicts 8 min of experiment; the playback speed is 60× real time.
